# A new risk score to assess atrial fibrillation risk in hypertensive patients (ESCARVAL-RISK Project

**DOI:** 10.1038/s41598-020-61437-w

**Published:** 2020-03-16

**Authors:** Domingo Orozco-Beltran, Jose A. Quesada, Vicente Bertomeu-Gonzalez, Jose M. Lobos-Bejarano, Jorge Navarro-Perez, Vicente F. Gil-Guillen, Luis Garcia Ortiz, Adriana Lopez-Pineda, Angel Castellanos-Rodriguez, Angela Lopez-Domenech, Antonio Francisco J. Cardona-Llorens, Concepcion Carratala-Munuera

**Affiliations:** 1 Chair of Family Medicine, Clinical Medicine Department, Miguel Hernandez University of Elche, San Juan de Alicante, Spain; 2 Cardiology Department, University Hospital of San Juan de Alicante, San Juan de Alicante, Spain; 3 Clinical Medicine Department, Miguel Hernandez University, San Juan de Alicante, Spain; 4 CIBER Cardiovascular CB16/11/00420, Madrid, Spain; 5 Jazmin Primary Care Health Center, Madrid, Spain; 6 Department of Internal Medicine, Valencia Clinical Hospital, Valencia, Spain; 7 INCLIVA Research Institute, Valencia, Spain; 8 Department of Medicine, University of Valencia, Valencia, Spain; 9 Cardiovascular Research Group of Castilla y León, Health Center La Alamedilla de Salamanca, Salamanca, Spain. Research Network in Preventive Activities and Health Promotion (REDIAPP). Department of Biomedical and Diagnostic Sciences, University of Salamanca, Salamanca, Spain; 10 Ciudad Periodistas Primary Care Health Center, Madrid, Spain; 11 Pathology and Surgery Department, Miguel Hernandez University of Elche, San Juan de Alicante, Spain

**Keywords:** Cardiology, Risk factors

## Abstract

This study aimed to assess atrial fibrillation (AF) incidence and predictive factors in hypertensive patients and to formulate an AF risk assessment score that can be used to identify the patients most likely to develop AF. This was a cohort study of patients recruited in primary healthcare centers. Patients aged 40 years or older with hypertension, free of AF and with no previous cardiovascular events were included. Patients attended annual visits according to clinical practice until the end of study or onset of AF. The association between AF incidence and explanatory variables (age, sex, body mass index, medical history and other) was analyzed. Finally, 12,206 patients were included (52.6% men, and mean age was 64.9 years); the mean follow-up was 36.7 months, and 394 (3.2%) patients were diagnosed with AF. The incidence of AF was 10.5/1000 person-years. Age (hazard ratio [HR] 1.06 per year; 95% confidence interval [CI] 1.05–1.08), male sex (HR 1.88; 95% CI 1.53–2.31), obesity (HR 2.57; 95% CI 1.70–3.90), and heart failure (HR 2.44; 95% CI 1.45–4.11) were independent predictors (p < 0.001). We propose a risk score based on significant predictors, which enables the identification of people with hypertension who are at the greatest risk of AF.

## Introduction

Data from the World Health Organization (WHO) show that 33.5 million people worldwide— or 0.5% of the world’s population—had atrial fibrillation (AF) in 2013, with higher prevalence detected in industrialized countries^1^. Indeed, in Europe and the United States, different studies have estimated a prevalence of 1% to 3%, depending on the population assessed and their age^2,3^. The demographic burden of AF is rising dramatically worldwide due to population ageing and probably to an increase in risk factors, particularly the obesity-diabetes pairing^4,5^.

In clinical practice, recognizing arrhythmia may be difficult, particularly in its subclinical and paroxysmal forms; these challenges explain a large part of the current underdiagnosis^5^. Often, AF is diagnosed incidentally in asymptomatic patients or in those with non-specific symptoms^6^. Previous studies have identified certain factors associated with a greater risk of developing AF^7,8^, opening the door to earlier detection through close monitoring of these patients. The Framingham Heart Study^9^ has reinforced prior knowledge of individual risk factors for AF, which include age, sex, body mass index (BMI), blood pressure variables, and prevalent cardiovascular disease. Moreover, that study and others^8,10^ have highlighted the role of obesity in AF, either as an independent precursor to AF or as a determinant linked to other more important risk factors such as type 2 diabetes and arterial hypertension. Other predictive factors include alterations detected during basal 12-lead electrocardiography (for example, P wave duration, PR interval or high density supraventricular extrasystolia) and echocardiography (for instance, left atrial dilatation)^9^.

AF is closely related to arterial hypertension, considered its main causal factor^11^ primarily because of its high prevalence^12^. Previous reports have observed that at least 75% of patients with AF who are attended in primary care are hypertensive^4,6^. Left ventricle hypertrophy and left atrial enlargement and remodeling are common underlying physiopathological features. Improved community control of hypertension has contributed to decreasing age-adjusted mortality for ischemic stroke^13^; however, hypertension and AF, alone or together, continue being the main risk factors for stroke^14^.

The ischemic strokes that are related to AF (at least one in four) are the most lethal, debilitating and costly in terms of social and healthcare resources^4^. Paradoxically, these cardioembolic events are more amenable to preventive anticoagulant therapy than those not associated with AF^15^, with meta-analyses showing reductions of up to 64%^16^. Moreover, AF may be responsible for a high proportion of cryptogenic stroke, which presents challenges for detecting arrhythmia and requires advanced diagnostic tools^17^. Thus, identifying predictors for AF and patients at risk could improve prevention of arrhythmia (for example, through behavioral modifications, blood pressure control, and—where appropriate—treatment with inhibitors of the renin-angiotensin-aldosterone system [RAAS]^18,19^).

Investigators involved in the Framingham Heart Study^9^, the Atherosclerosis Risk In Communities (ARIC) study^20^, and the Cohorts for Aging and Research in Genomic Epidemiology (CHARGE)-AF study^21^—all population-based cohort studies—have developed scoring systems for AF risk assessment, but none of these have been validated in people with hypertension despite this being the main risk factor for the disease^11^. Our prospective study aims to assess AF incidence and predictive factors in a cohort of hypertensive patients included in the ESCARVAL-RISK study (EStudio CARdiometabolico VALenciano)^22^ and to formulate an AF risk assessment scale that can be used in clinical practice to accurately identify the patients most likely to develop AF.

## Methods

### Study participants

This is an observational cohort study of patients who were consecutively recruited from January 1, 2007 to December 31, 2010 in public primary healthcare centers of the region of Valencia (population 4,980,689 according to the 2015 census), run by the Valencian Health Agency, with a public health system where all population has free access to primary or secondary care.

Inclusion criteria were: residents of the region of Valencia, with available medical records in the Electronic Record System (ABUCASIS); free of AF; aged 40 years or older; diagnosed with hypertension; and with no previous cardiovascular events who attended a primary healthcare center for routine visits (ESCARVAL-RISK cohort^22^). Diagnosis of hypertension was according to the following criteria: systolic blood pressure of at least 140 mmHg or diastolic blood pressure of at least 90 mmHg, as documented in two separate and consecutive readings; diagnosis according to ICD code 401.9; or receiving any medication for blood pressure. We excluded patients with any type of AF known prior to study commencement, history of high or moderate alcohol consumption, short life expectancy, or any other diseases, like hyperthyroidism and severe chronic obstructive pulmonary disease (COPD), that the attending physician believed could skew study results. Other mental or social factors that could present challenges for follow-up were also exclusion criteria, as was the unavailability of valid indicators for the analysis variables. Patients who presented a cardiovascular event during follow up were not excluded. Our inclusion criteria, study variables, and methodology have been published in detail elsewhere^22^.

The Committee for Ethics and Clinical Trials (CEIC in its Spanish abbreviation) of the Center for Public Health and Public Health Research (DGSP-CSISP in its Spanish abbreviation) approved the study protocol, and participants provided written informed consent. The repository of data is not available. The study was performed in accordance with relevant guidelines and regulations.

### Study variables

Our response variable was the appearance (yes/no) of AF. Explanatory variables were age; sex; body mass index (BMI: normal, <25 kg/m^2^; overweight, 25–30 kg/m^2^; grade I obesity, 30–35 kg/m^2^; and grade II obesity, >35 kg/m^2^); tobacco use (non-smoker/smoker or ex-smoker); history of diabetes mellitus (yes/no) and/or dyslipidemia (yes/no), onset of ischemic heart disease (IHD) (yes/no), stroke (yes/no), and/or congestive heart failure (CHF) (yes/no) during follow-up; treatment with antihypertensive therapy (yes/no) or for diabetes (yes/no), dyslipidemia (yes/no), and/or hypertension (yes/no); and analytical variables like systolic and diastolic blood pressure, fasting glucose levels, total cholesterol, high-density lipoprotein (HDL) cholesterol, low-density lipoprotein (LDL) cholesterol, triglycerides, serum creatinine, and estimated glomerular filtration rate (eGFR) as calculated with the CKD-EPI creatinine equation^23^.

### Follow-up

At the initial visit, physicians collected data on study variables. Thereafter, participants attended annual visits to undergo a thorough examination for AF; follow-up checks continued until the end of study or onset of AF. In addition, all patients with symptoms were further studied in the cardiology departments, including by echocardiography and ambulatory electrocardiography monitoring. Diagnosis was based on electrocardiographic readings according to the criteria of the European Society of Cardiology^24^ and documented in the participant’s medical record (ICD-code 427.3).

### Statistical analysis

We performed a bivariate analysis to test the association between AF incidence and the qualitative explanatory variables using contingency tables, calculating the P value with the chi-square test of association. For quantitative variables, we determined the minimum, maximum, mean, and standard deviation, and we compared the mean value according to presence/absence of AF using the student’s t test. We adjusted the estimated risk of AF using the Cox proportional hazards model, estimating the hazard ratio (HR) and its 95% confidence interval (CI). We employed backward selection of variables according to the Akaike information criterion (AIC) and verified the fit of the model using the likelihood ratio test (LRT), then estimating the C-index and its 95% CI as a measure of prediction. We analyzed the possible interactions between variables and tested the Cox proportional hazards assumption. The c-index with 95% confidence intervals was calculated using 10-fold cross-validation (repeated 30 times and averaged). Finally, we formulated a table of risks and probabilities of developing AF over three years, using the optimal model based on the points system described by Sullivan *et al*. in 2004^25^. For statistical analyses, we used SPSS software (version 18.0) and the R package (version 3.2.3).

## Results

Our final sample size included 12,206 participants; 52.6% were men, and mean age was 64.9 years (range 40 to 94). Longest follow-up was 47 months (mean 36.7).

**Figure 1:**
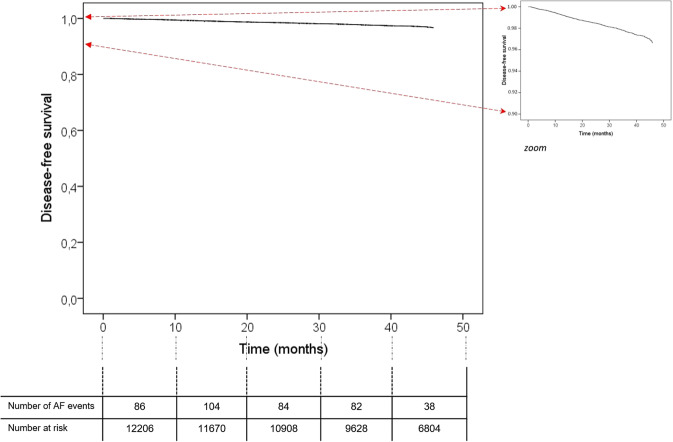
Arrhythmia-free survival, according to the multivariate model.

Table 1 shows the incidence of AF at study end, according to qualitative indicators for the overall study cohort. At the end of the study period, 394 (3.2%) participants had been diagnosed with AF (Fig. 1). Incidence was 10.5 per 1000 person-years (12.9/1000 person-years in men and 8.0/1000 person-years in women). Qualitative variables that were significantly associated with incident AF comprised age; sex; BMI; history of diabetes mellitus, onset of IHD, stroke or CHF; and treatment for diabetes. The highest incidence occurred in participants with a history of CHF, at 10.8% (Table 1). Quantitative factors that showed a significant association with AF were diastolic blood pressure, total cholesterol, LDL cholesterol, triglycerides, serum creatinine and eGFR (Table 2).

**Table 1:** Incidence of AF at study end, according to qualitative indicators (chi-square test).

Participant characteristics		Total		No AF		Yes AF		*P* value
		n	%	n	%	n	%	
Age	<60 years	3636	29.8	3596	98.9	40	1.1	<0.001*
	60–74 years	6280	51.5	6065	96.6	215	3.4	
	≥ 75 years	2290	18.8	2151	93.9	139	6.1	
Sex	Men	6416	52.6	6168	96.1	248	3.9	<0.001*
	Women	5790	47.4	5644	97.5	146	2.5	
BMI	Normal <25 kg/m^2^	1278	10.5	1247	97.6	31	2.4	0.002*
	Overweight 25–30 kg/m^2^	5050	41.4	4896	97.0	154	3.0	
	Grade I obesity, 30–35 kg/m^2^	4091	33.5	3965	96.9	126	3.1	
	Grade II obesity > 35 kg/m^2^	1787	14.6	1704	95.4	83	4.6	
Smoking	Non-smoker	6880	56.4	6658	96.8	222	3.2	0.993
	Smoker/ex-smoker	5326	43.6	5154	96.8	172	3.2	
Diabetes mellitus	No	8399	68.8	8158	97.1	241	2.9	0.001*
	Yes	3807	31.2	3654	96.0	153	4.0	
Dyslipidemia	No	6999	57.3	6767	96.7	232	3.3	0.529
	Yes	5207	42.7	5045	96.9	162	3.1	
IHD	No	11628	95.3	11271	96.9	357	3.1	<0.001*
	Yes	578	4.7	541	93.6	37	6.4	
Stroke	No	11931	97.7	11,552	96.8	379	3.2	0.035*
	Yes	275	2.3	260	94.5	15	5.5	
CHF	No	12067	98.9	11,688	96.9	379	3.1	<0.001*
	Yes	139	1.1	124	89.2	15	10.8	
BP treatment	No	7462	61.1	7233	96.9	229	3.1	0.212
	Yes	4744	38.9	4579	96.5	165	3.5	

AF: atrial fibrillation; BMI: body mass index; BP: blood pressure; IHD: ischemic heart disease; CHF: congestive heart failure.

*Statistically significant (*P* < 0.05).

**Table 2:** Quantitative variables, according to incidence of atrial fibrillation (student’s t test).

	AF	n	Minimum	Maximum	Mean	SD	*P* value
Age (years)	No	11812	40.0	94.0	64.7	10.3	<0.001*
	Yes	394	41.0	91.0	70.7	8.6	
BMI (kg/m^2^)	No	11812	15.2	68.0	30.3	4.8	<0.001*
	Yes	394	18.3	55.9	31.3	5.5	
Diastolic blood pressure (mmHg)	No	11642	40.0	140.0	80.6	11.3	<0.001*
	Yes	387	46.0	140.0	77.5	11.8	
Systolic blood pressure (mmHg)	No	11640	68.0	240.0	141.6	18.9	0.436
	Yes	387	95.0	220.0	140.9	20.4	
Total cholesterol (mg/dL)	No	4937	81.0	397.0	204.4	37.2	0.050*
	Yes	142	119.0	305.0	198.2	36.1	
HDL cholesterol (mg/dL)	No	4564	15.0	100.0	51.5	13.6	0.396
	Yes	130	25.0	96.0	50.5	13.9	
LDL cholesterol (mg/dL)	No	3550	60.0	150.0	115.4	22.2	0.029*
	Yes	104	61.0	150.0	110.6	24.4	
Triglycerides (mg/dL)	No	4457	55.0	397.0	142.8	67.6	0.005*
	Yes	133	55.0	380.0	128.9	54.5	
Serum creatinine (mg/dL)	No	4105	0.50	6.00	0.94	0.29	0.006*
	Yes	120	0.55	3.10	1.01	0.32	
eGFR^†^ (ml/min/1.73 m^2^)	No	4105	7.0	124.2	79.7	17.7	<0.001*
	Yes	120	19.5	104.2	71.3	17.4	
Glucose (mg/dL)	No	4973	61.0	299.0	117.5	36.9	0.215
	Yes	139	68.0	298.0	121.4	38.2	

AF: atrial fibrillation; BMI: body mass index; eGFR: estimated glomerular filtration rate; HDL: high-density lipoprotein; LDL: low-density lipoprotein; SD: standard deviation.

*Statistically significant (P < 0.05).

^†^Measured by means of CKD-EPI creatinine equation.

The multivariate Cox model fitted the data well, presenting an LRT value of 181.3 (*P* < 0.001). Predictive value was acceptable, with a C-index of 0.69 (95% CI 0.66, 0.72). All the variables in the model met the proportional hazards assumption. We did not detect significant interaction between any of the model’s variables.

Significant predictors of AF were age, BMI, sex, and CHF. The factor associated with the highest risk was grade II obesity (HR 2.57, 95% CI 1.67, 3.90), followed by CHF (HR 2.44, 95% CI 1.45, 4.11) (Table 3). Based on the data obtained, we assigned a point score for each qualitative and quantitative risk factor, with the sum reflecting the probability of developing AF within three years (Tables 4 and 5). Figure 1 shows the arrhythmia-free survival, according to multivariate model. The survival rate at the end of the study period was 96.6%.

**Table 3:** Multivariate Cox regression model for incidence of atrial fibrillation. Cox proportional hazards regression coefficients for direct estimation of risk of first AF event.

	β	SE	HR	95% CI		*P* value
Normal BMI < 25 kg/m^2^	0		1			
Overweight 25–30 kg/m^2^	0.22184	0.197	1.25	0.85	1.84	0.261
Grade I obesity, 30–35 kg/m^2^	0.31609	0.201	1.37	0.93	2.04	0.116
Grade II obesity > 35 kg/m^2^	0.94324	0.214	2.57	1.70	3.90	<0.001*
Age	0.06187	0.006	1.06	1.05	1.08	<0.001*
Women	0		1			
Men	0.63036	0.106	1.88	1.53	2.31	<0.001*
No CHF	0		1			
CHF	0.89367	0.265	2.44	1.45	4.11	0.001*

β: standardized coefficient; BMI: body mass index; CHF: congestive heart failure; CI: confidence interval; HR: hazard ratio; SE: standard error of the coefficient.

*Statistically significant (P < 0.05).

**Table 4:** Risk score for each category of variables in multivariate model.

Risk factor	Categories	Points
Age (years)	40–44	−2
	45–49	−1
	50–54	0
	55–59	1
	60–64	2
	65–69	3
	70–74	4
	75–79	5
	80–84	6
	85–89	7
	90–94	8
Sex	Women	0
	Men	2
BMI	<25 kg/m^2^ normal	0
	25–30 kg/m^2^ overweight	1
	30–35 kg/m^2^ grade I obesity	1
	>35 kg/m^2^ grade II obesity	3
CHF	No	0
	Yes	3

BMI: body mass index; CHF: congestive heart failure.

**Table 5:** Probability of developing atrial fibrillation within three years, according to risk score.

Score	Estimated risk
≤ 0	<0.5%
1	0.7%
2	1.0%
3	1.3%
4	1.8%
5	2.5%
6	3.3%
7	4.5%
8	6.1%
9	8.3%
10	11.1%
11	14.8%
12	19.6%
13	25.7%
14	33.3%
15	42.4%
16	52.8%

## Discussion

### Main findings

We observed a mean incidence of 10.5 cases of AF per 1000 person-years in hypertensive patients over the age of 40, with more cases appearing in men than in women. The multivariate Cox regression model showed that significant predictors for AF include age, male gender, BMI, and CHF. Based on these findings and indicators, we developed a three-year risk score for incident AF in a prospective, population-based cohort with hypertension.

### Comparison with other studies

The incidence of AF in this hypertensive study cohort was higher than that reported at 10 years in the general population in the Framingham Heart Study (6.3 per 1000 age-adjusted person-years in men and 3.3 per 1000 age-adjusted person-years in women)^9^. Previous studies in Europe^26,27^, also in the general population, have likewise observed lower incidence rates than in our study. However, other authors have noted that the risk of developing AF is significantly higher in people with hypertension compared to people with normal blood pressure^28^. In 2482 hypertensive patients with a mean age of 51 years and no initial pharmacological treatment, Verdecchia *et al*.^19^ found an AF rate of 4.6 per 1000 person-years. In contrast, Alvez-Cabratosa *et al*.^29^ observed an incidence rate that is more consistent with ours, of 12.5 per 1000 person-years in hypertensive patients in Spain. With regard to incidence by age group and sex, our results are concordant with those published elsewhere^26,27^: incidence is higher in men than in women and increases with age.

In addition, and similarly to previous studies^4,7^, we found several qualitative and quantitative variables associated with the appearance of AF. In line with other studies over the past decade^8,10^, obesity stands out as a major independent predictor, with grade II obesity conferring a similar level of AF risk as CHF. However, CHF is not always an etiological factor in AF, as the causal pathway between the two conditions goes in both directions^30^. They often share underlying features constituting the causal mechanism, such as hypertension and ischemic heart disease, whose optimal medical treatment can prevent or delay the appearance of both arrhythmia and CHF^30^.

Obesity is attracting increased attention as a risk factor for AF, based on epidemiological, physiopathological, and clinical evidence^31,32^. Obesity is associated with diastolic dysfunction; a systemic proinflammatory state; atrial dilatation; and the presence of metabolically active pericardial adipose tissue, which produces arrhythmogenesis^33^. Moreover, obesity is closely linked to other cardiometabolic risk factors (diabetes, dyslipidemia, and hypertension) and to other well-known determinants of AF (e.g. obstructive sleep apnea)^10,31^. In the OFRECE study^34^, the presence of obesity and central obesity increased the likelihood of developing AF more than any other cardiovascular risk factor. Unlike other predictors, obesity is also a modifiable risk factor, as reported by Abed *et al*.^31^ and Pathak *et al*.^32^. Thus, in people with AF, non-pharmacological interventions to reduce weight and waist circumference (basically modifications of diet and physical activity), together with optimal treatment for cardiometabolic risk factors, can lead to proportional reductions in the severity of symptoms and the necessity to use antiarrhythmic drugs. The LEGACY study^32^ in Australia followed 355 patients with paroxysmal or persistent AF and a BMI of 27 kg/m^2^ or more (78% were hypertensive) for five years; investigators found that reducing baseline body weight by 10% or more was associated with a six-fold greater probability of arrhythmia-free survival.

Other studies have reported that antihypertensive treatment can reduce the risk of AF in people with hypertension^35,36^; however, our results did not confirm this finding, probably because the study took place in the context of routine clinical practice. In this setting, clinical inertia and lack of therapeutic adherence are commonplace^37^, and in our population there was a high proportion of patients not being treated for hypertension despite their medical records showing high blood pressure—either because they had not received a diagnosis for hypertension or because their clinician had only recommended behavioral modifications, with a more dubious potential impact.

Finally, our proposed risk score showed an acceptable predictive capacity (C-index = 0.69), although lower than those obtained in the original cohorts from the general population of Framingham^9^ or ARIC^20^, both yielding a C-index of 0.78; or the more recent CHARGE-AF model^21^ (C-index = 0.77). However, the C-index in these studies has tended to decrease when used in external populations; for example, the C-index for the Framingham risk score was 0.68 when validated in the ARIC study population. All these scores have been developed in American cohorts, which have proven very different from Mediterranean patients. Furthermore, all of these scoring systems use a greater number of variables than we do; the CHARGE-AF model considers age, race, height, weight, systolic and diastolic blood pressure, current smoking, use of antihypertensive medication, diabetes, and history of myocardial infarction and CHF.

### Strengths and limitations

There are few predictive risk scores for AF and to our knowledge, this is the only one that specifically focuses on patients with hypertension. However, readers should consider the results of this observational study in light of the advantages and limitations of registry-based data. On the one hand, electronic health records provide quick and low-cost access to rich longitudinal data on large populations for epidemiologic research. Moreover, registry data are a good reflection of real clinical practice. On the other hand, there is also a greater chance of AF not being properly recorded in routine clinical practice. In order to validate the correct interpretation of electrocardiogram by physicians, an audit was performed in 10% of patients with AF at the end of the study, i.e. we checked the electrocardiogram results in 10% of patients with AF, and 100% were correct. Regarding the variables collected, sleep apnea, physical inactivity and high-intensity exercise increase AF risk; however, these variables are not routinely recorded in medical history by primary care physicians, so we could not include them in the study analysis. The echocardiography is also of great value to predict atrial fibrillation in individuals, but it is not a good variable to include in a risk score due to its low availability. Another potential limitation is that our risk score is derived from a single, predominantly white Mediterranean hypertensive cohort. Some authors have reported that the most commonly used risk scores, like Framingham, overestimate high risk in Mediterranean populations^38^, potentially leading to many overtreated patients. Thus, further research in needed to validate the findings in a different cohort.

### Implications

Researchers and clinicians have always valued the simplicity of a risk score for clinical practice, even at the expense of accuracy. However, nowadays all medical histories are electronic, so it is possible to achieve a more elaborate and accurate risk score without sacrificing ease of use. Furthermore, other AF risk scores have not been validated in a Mediterranean population. Further research is needed to compare the predictive capacity of our risk score versus other AF risk scores in this context. The present study demonstrates that a few simple indicators—easily obtained in clinical examination, like age, sex, BMI and history of CHF—are independently associated with a higher incidence of AF in a Mediterranean population. Clinicians may consider these predictors during the consultation and perform simple follow-up of hypertensive patients in clinical practice: (i) facilitating early detection of AF through opportunistic screening, pulse measurement, and eventually electrocardiogram, through 24-hour electrocardiographic (Holter) monitoring or even through pulse monitoring using wearable health devices; (ii) intensifying the control of blood pressure; (iii) enabling the preferential use of medications that can delay the appearance of arrhythmia (RAAS inhibitors); and (iv) intervening energetically to modify behaviors, particularly sedentarism and obesity. Finally, the proposed risk score could entail improvements in the prevention and early detection of AF in the hypertensive population. All these improvement strategies should be validated in studies specifically designed for this purpose.

## Conclusions

Incidence of atrial fibrillation was 10.5 cases per 1000 person-years among a cohort of people with hypertension attending primary care. Age, sex, obesity and CHF were independently predictive of AF. We propose a simple risk score based on these four readily available variables, which enables the identification of people with hypertension who are at the greatest risk of AF, and subsequently the implementation of effective interventions to address modifiable risk factors.
