# Author Correction: A new risk score to assess atrial fibrillation risk in hypertensive patients (ESCARVAL-RISK Project

**DOI:** 10.1038/s41598-023-40306-2

**Published:** 2023-08-16

**Authors:** Domingo Orozco-Beltran, Jose A. Quesada, Vicente Bertomeu-Gonzalez, Jose M. Lobos-Bejarano, Jorge Navarro-Perez, Vicente F. Gil-Guillen, Luis Garcia Ortiz, Adriana Lopez-Pineda, Angel Castellanos-Rodriguez, Angela Lopez-Domenech, Antonio Francisco J. Cardona-Llorens, Concepcion Carratala-Munuera

**Affiliations:** 1 Chair of Family Medicine, Clinical Medicine Department, Miguel Hernandez University of Elche, San Juan de Alicante, Spain; 2 Cardiology Department, University Hospital of San Juan de Alicante, San Juan de Alicante, Spain; 3 Clinical Medicine Department, Miguel Hernandez University, San Juan de Alicante, Spain; 4 CIBER Cardiovascular CB16/11/00420, Madrid, Spain; 5 Jazmin Primary Care Health Center, Madrid, Spain; 6 Department of Internal Medicine, Valencia Clinical Hospital, Valencia, Spain; 7 INCLIVA Research Institute, Valencia, Spain; 8 Department of Medicine, University of Valencia, Valencia, Spain; 9 Cardiovascular Research Group of Castilla Y León, Health Center La Alamedilla de Salamanca, Salamanca, Spain. Research Network in Preventive Activities and Health Promotion (REDIAPP). Department of Biomedical and Diagnostic Sciences, University of Salamanca, Salamanca, Spain; 10 Ciudad Periodistas Primary Care Health Center, Madrid, Spain; 11 Pathology and Surgery Department, Miguel Hernandez University of Elche, San Juan de Alicante, Spain

Correction to: *Scientific Reports* 10.1038/s41598-020-61437-w, published online 16 March 2020

The original version of this Article contained an error in the Acknowledgements section. The last digit of the RETICS was incomplete.

“This study was funded by the Spanish Ministry of Science and Innovation (MICINN) and Carlos III Health Institute (ISCIII)/European Regional Development Fund (ERDF), (RETICS: RD16/0007). The authors declare no conflicts of interest.”

now reads:

“This study was funded by the Spanish Ministry of Science and Innovation (MICINN) and Carlos III Health Institute (ISCIII)/European Regional Development Fund (ERDF), (RETICS: RD16/0007/0015). The authors declare no conflicts of interest.”

The original Article has been corrected.

